# Pretreatment prostate-specific antigen density as a predictor of biochemical recurrence in patients with prostate cancer: a meta-analysis

**DOI:** 10.1186/s12885-024-12029-8

**Published:** 2024-03-06

**Authors:** Feilun Cui, Yue Qiu, Wei Xu, Chen Zou, Yu Fan

**Affiliations:** 1 Department of Urology, Affiliated Taizhou Second People’s Hospital of Yangzhou University , 225500 Taizhou, China; 2 Department of Urology, The Fourth Affiliated Hospital of Jiangsu University , 212002 Zhenjiang, China; 3 Cancer Institute, The Affiliated People’s Hospital, Jiangsu University, No. 8 Dianli Road, 225500 Zhenjiang, Zhenjiang China; 4 Department of General Surgery, Suzhou Hospital, Affiliated Hospital of Medical School Nanjing University, No. 1 Lijiang Road, 215163 Suzhou, China

**Keywords:** Prostate-specific antigen density, Biochemical recurrence, Prostate cancer, meta-analysis

## Abstract

**Background:**

A consensus has not been reached on the value of prostate-specific antigen density (PSAD) as a predictor of biochemical recurrence of prostate cancer. This meta-analysis aimed to evaluate the association between PSAD and biochemical recurrence of prostate cancer after primary treatment.

**Methods:**

Two authors systematically searched PubMed, Web of Science, and Embase databases (up to August September 10, 2023) to identify studies that assessed the value of pretreatment PSAD in predicting biochemical recurrence after primary treatment (radical prostatectomy or radiotherapy) of prostate cancer. A random effect model was used to pool adjusted hazard ratios (HR) with 95% confidence intervals (CI) for biochemical recurrence.

**Results:**

Nine studies with 4963 patients were eligible for the meta-analysis. The reported prevalence of biochemical recurrence ranged from 4 to 55.1%. For patients with higher PSAD compared to those with low PSAD, the pooled HR of biochemical recurrence was 1.59 (95% CI 1.21–2.10). Subgroup analysis showed that the pooled HR of biochemical recurrence was 1.80 (95% CI 1.34–2.42) for patients who received radical prostatectomy, and 0.98 (95% CI 0.66–1.45) for patients who received radiotherapy.

**Conclusions:**

Elevated pretreatment PSAD may be an independent predictor for biochemical recurrence of prostate cancer after radical prostatectomy. Determining PSAD could potentially improve the prediction of biochemical recurrence in patients with prostate cancer.

**Supplementary Information:**

The online version contains supplementary material available at 10.1186/s12885-024-12029-8.

## Background

Prostate cancer is the second most common type of cancer and the fifth leading cause of mortality among men [1]. Worldwide, there were approximately 1,414,259 newly diagnosed cases and 375,304 deaths from prostate cancer reported in 2020 [2]. Biochemical recurrence (BCR) after primary curative treatment may may indicate a more advanced or aggressive form of the disease. Almost one-third of men with prostate cancer experience BCR after primary treatment [3]. BCR is considered a marker for local recurrence, distant metastasis, and prostate-specific survival [4]. Determining BCR after primary treatment can help identify treatment failure and determine the need for salvage therapy. Therefore, improving the risk stratification of BCR is critical for better management of prostate cancer patients.

Prostate-specific antigen (PSA), a kallikrein-related serine protease, is widely used for prostate cancer screening. However, the blood PSA level can be affected by the size of the prostate gland. Prostate-specific antigen density (PSAD) is typically calculated by determining the ratio between the blood PSA level (ng/mL) and the estimated prostate volume (cm^3^) before treatment. Initially, PSAD was used to differentiate between benign prostatic disease and prostate cancer [5]. Subsequently, has been studied as a potential indicator for adverse pathological features [6, 7] or BCR [8, 9] after primary prostate cancer treatment. However, there is still conflicting evidence regarding whether pretreatment PSAD can independently predict BCR in patients with prostate cancer [8–14].

No previous meta-analysis has been conducted to investigate the association of PSAD with BCR of prostate cancer to date. Consequently, we conducted the present meta-analysis to further elucidate the significance of pretreatment PSAD as a prognostic factor for BCR in patients with prostate cancer.

## Materials and methods

### Study guideline and ethics approval

This study was prepared according to the checklist of the Preferred Reporting Items for Systematic Reviews and Meta-Analyses [15]. Ethical approval was not necessary as the study did not involve individual patient data.

### Literature search

Two authors conducted a thorough search on PubMed, Web of Science, and Embase databases until August September 10, 2023. The search utilized the following keywords (Supplemental Text S1): (“prostate neoplasms” OR “prostate cancer” OR “prostate tumor” OR “prostate carcinoma”) AND (“prostate-specific antigen density” OR “PSAD”) AND (“biochemical recurrence” OR “biochemical failure” OR “relapse”). Additionally, the authors manually reviewed references from included studies and relevant reviews for potential inclusion.

### Study selection

Two authors independently evaluated the eligibility of the retrieved studies using the following criteria. The inclusion criteria included:1) patients with a diagnosis of prostate cancer who received radical prostatectomy or radiotherapy, 2) pretreatment PSAD level as a predictor,3) BCR defined as at least two consecutive PSA level elevation after primary curative treatment as the outcome of interest, 4) reported multivariable adjusted risk estimates of BCR for the categorical analysis of PSAD, and 5) prospective or retrospective observational study as the design. The cutoff value of PSAD elevation was defined by the individual study. The criteria for exclusion were as follow: (1) studies lacking risk estimate data, (2) reporting unadjusted risk estimates, and (3) reporting risk estimate by continuously coding PSAD.

### Data extraction and quality assessment

Using a pre-designed data extraction form, two independent authors abstracted the following information: surname of the first author, year of publication, country of origin, design of study, type of prostate cancer, baseline age of the patients, number of patients, treatment approach, cutoff category of PSAD, definition of BCR, percentage of patients with BCR, follow-up duration, adjusted risk estimate, and adjusted confounders. The methodological quality of included studies was assess by two independent authors using the Newcastle-Ottawa Scale (NOS) [16]. The studies with the total score 7 or higher were considered high-quality. Disagreements in the data extraction and quality evaluation process were resolved by consensus.

### Statistical analysis

All analyzes were conducted with Stata version12.0 (Stata Corporation, College Station, TX). The prognostic value of PSAD for BCR was summarized by combining adjusted hazard ratios (HR) with 95% confidence intervals (CI) for high vs. low PSAD category. The degree of heterogeneity across studies was evaluated using the I2 statistics and Cochran Q test. Statistical heterogeneity was determined by a *p*-value < 0.1 for the Cochran Q test and/or an *I*^2^ statistic greater than 50%. If statistical heterogeneity was present, a random effects model was used for the meta-analysis. To assess the reliability of the pooled summary, a sensitivity analysis was performed by excluding individual studies from the overall analysis each turn. Subgroup analyses were conducted based on the risk of patients according to the D’Amico criteria [17], country of origin, treatment approach, number of patients, cutoff value of PSAD, follow-up duration, and whether adjusted pretreatment PSA level. Funnel plots, Begg’s rank correlation test [18], and Egger’s linear regression test [19] were used to evaluate publication bias.

## Results

### Search results and characteristics of included studies

**Fig. 1:**
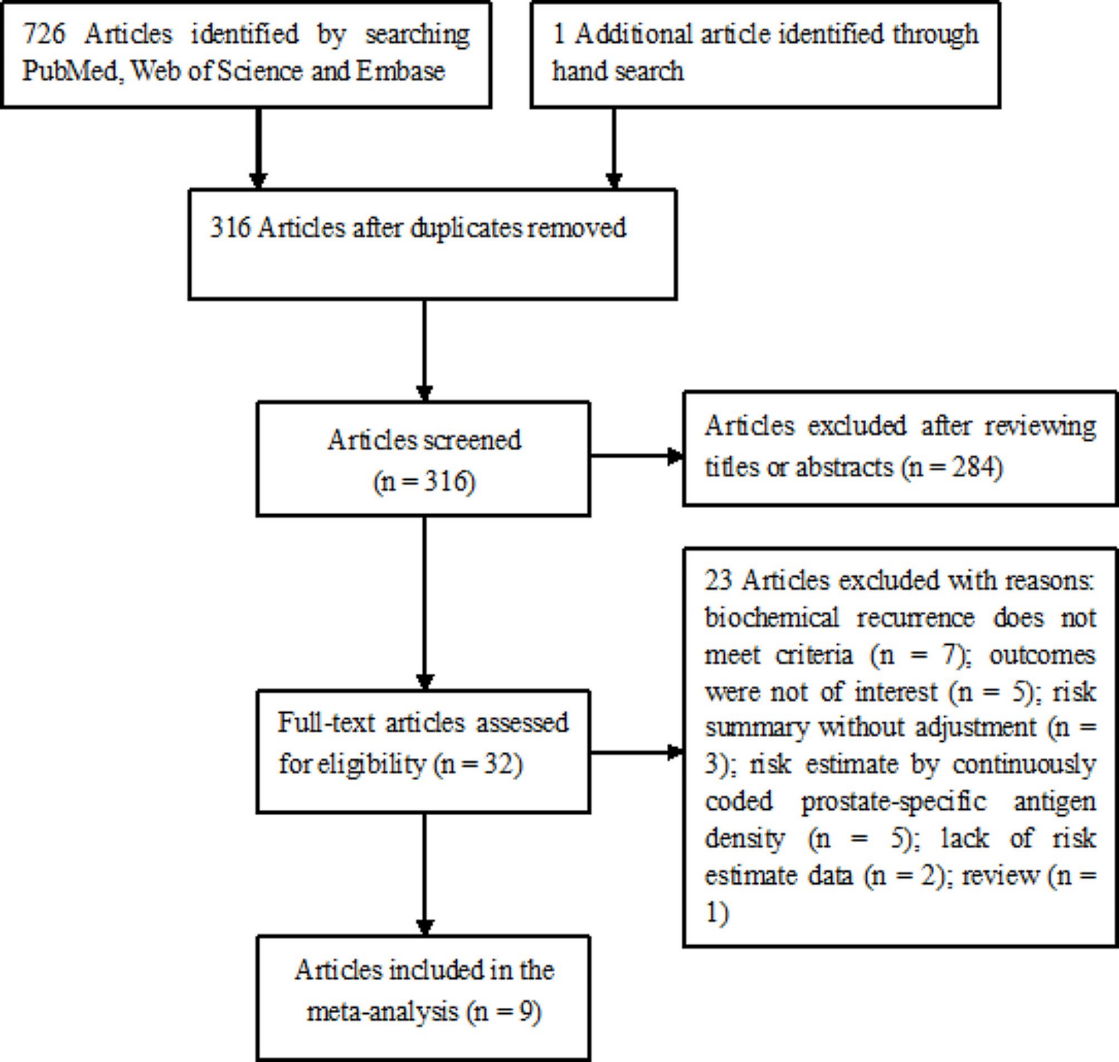
Flow chart of studies selection process

Figure 1 presents the meticulous process of study selection. Initially, a total of 727 publications were identified through our literature search. After eliminating duplicates, 316 articles remained for evaluation of their titles and abstracts. Of these, 32 full-text articles were retrieved for eligibility assessment. Following the application of predetermined inclusion and exclusion criteria, 23 articles were excluded. Finally, 9 studies [8, 9, 11–14, 20–22] were included in this meta-analysis.

Table 1 presents the baseline characteristics of the included studies. These studies were published between 1997 and 2022 and were all retrospective in nature. The eligible studies were conducted in various regions including Europe [11, 12], USA [8], Canada [9], Japan [13, 14, 21], and China [20, 22]. The sample sizes of individual studies ranged between 95 and 1334, resulting in a total of 4963 prostate cancer patients. The follow-up duration varied between 21 and 60.3 months. The reported prevalence of BCR ranged from 4 to 55.1%. The median/mean age of the patients ranged from 63 to 69.5 years old from studies reporting such data. Based on the NOS criteria, 2 studies [14, 21] were graded as moderate quality, while the remaining studies were graded as high-quality (Table S1).

**Table 1:** Main characteristics of included studies

Author/year	Region	Patients	Age (years)	Treatment	Cutoff value of PSAD	Definition of BCR (%)	Follow-up (months)	HR (95% CI)	Adjusted covariates
Ingenito 1997 [8]	USA	Localized PCa 175	NP	Radiotherapy	≥ 0.29 vs.<0.29	Three consecutive rise PSA (49)	25.2	1.20 (0.62–2.32)	Gleason score, pretreatment PSA
Aref 1998 [9]	Canada	Localized PCa 205	NP	Radiotherapy	≥ 0.3 vs.<0.3	Three consecutive rise PSA (55.1)	45	0.87 (0.53–1.42)	Age, clinical stage, Gleason score, pretreatment PSA
Busch 2012 [11]	Germany	PCa 1334	63 (43–75)	RP	≥ 0.22 vs.<0.22	Two rise PSA ≥ 0.1 ng/ml (18.1)	60.3	1.47(1.13–1.92)	Gleason sum, pathological stage, margin status, pretreatment PSA
Gandaglia 2015 [12]	Europe	Very Low-risk PCa 1710	63.9 (59–68)	RP	≥ 10 vs.<10	Two consecutive PSA ≥ 0.2 ng/ml (4.0)	40	1.68 (1.02–2.88)	Age at surgery, pretreatment PSA, clinical stage, number of cores taken, number of positive cores
Hashimoto 2015 [13]	Japan	Localized PCa 784	64.3 (60–69)	RP	≥ 0.4 vs.<0.4	Two consecutive PSA ≥ 0.2 ng/ml (10.2)	22.3	2.19 (1.37–3.50)	Age, percent positive core, clinical stage, Gleason score, lymph node metastasis, surgical margin
Yashi 2017 [14]	Japan	High-risk PCa 95	67 (63–71)	RP	> 0.345 vs. ≤0.345	PSA ≥ 0.1 ng/ml with subsequent rising PSA (27.4)	24.5	3.10(1.37–7.01) #	positive cores, dominant side, cancer extent
Peng 2019 [20]	China	Intermediate- risk PCa 169	68 (50–78)	RP	> 0.3 vs. ≤0.3	Two consecutive PSA ≥ 0.2 ng/ml (21.3)	31	3.07(1.38–6.82) #	Age, biopsy Gleason pattern, preoperative PSA, prostate volume, positive biopsies, number of intermediate risk factors, surgical margins, extracapsular tumor extension, seminal vesicle invasion,
Shida 2022 [21]	Japan	High-risk PCa 107	50–83	RP	> 0.5 vs. ≤0.5	Two consecutive PSA ≥ 0.2 ng/ml (26.2)	21	2.48(1.15–5.35)	pretreatment PSA, biopsy Gleason score, clinical stage, positive cores
Yan 2022 [22]	China	PCa 384	69.5 ± 6.5	RP	≥ 0.52 vs.<0.52	Two consecutive PSA ≥ 0.2 ng/ml (20.6)	41	0.92 (0.54–1.56)	Biopsy Gleason pattern, preoperative PSA, clinical stage, surgical margins, lymph node, seminal vesicle invasion, nerve invasion

Abbreviations: HR, hazard ratio; CI, confidence interval; NP, not provided; R, retrospective; PCa, prostate cancer; PSA, prostate-specific antigen; PSAD, prostate-specific antigen density; BCR, biochemical recurrence; RP, radical prostatectomy

### Association of PSAD with BCR

**Fig. 2:**
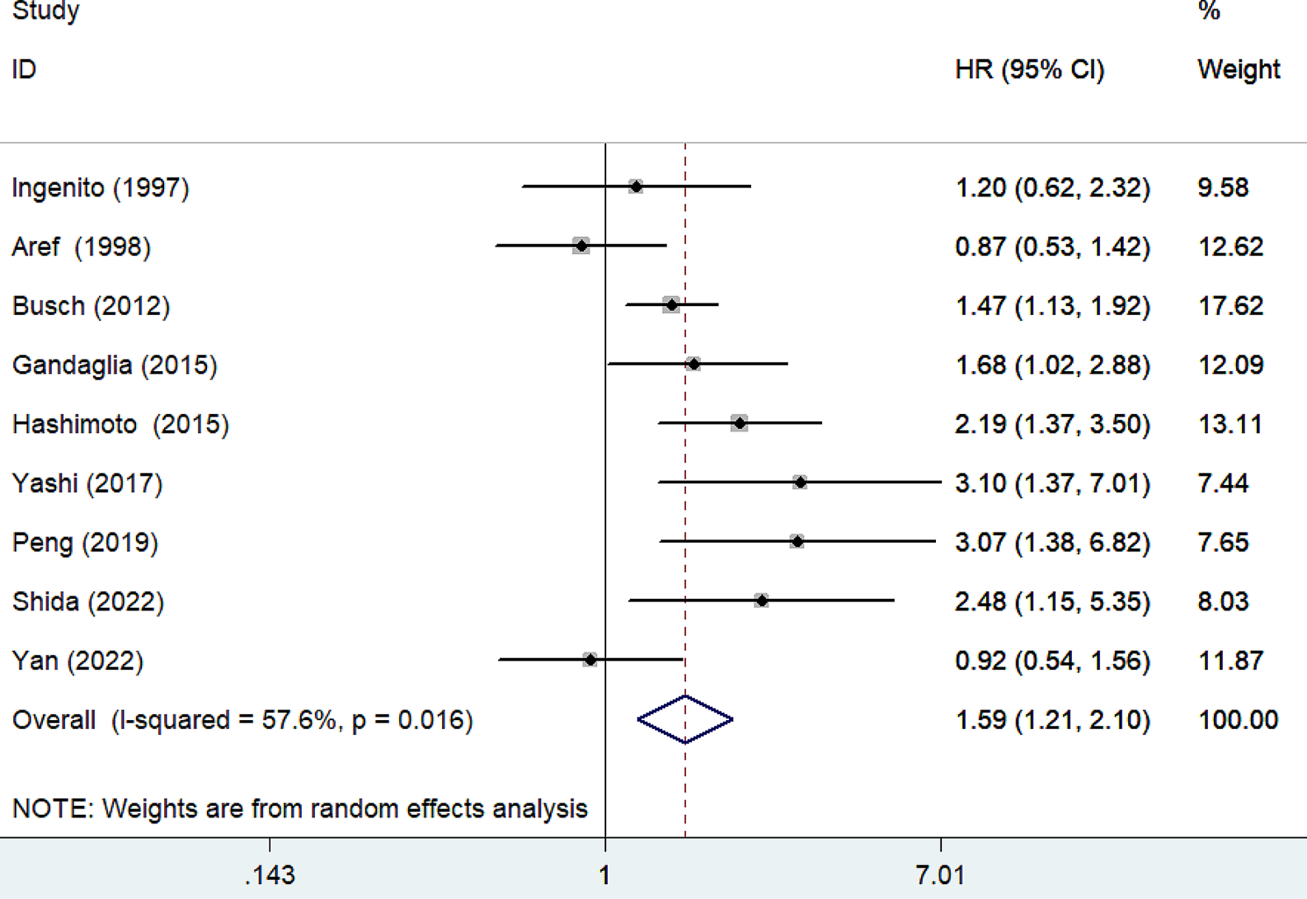
Forest plots showing pooled HR of biochemical recurrence for high versus low prostate-specific antigen density

As shown in Fig. 2, a meta-analysis using a random effect model showed that the pooled adjusted HR for BCR was 1.59 (95% CI 1.21–2.10) in the high PSAD category compared to the low category. There was significant heterogeneity (*I*^2^ = 57.6%; *p* = 0.016) across studies. Leave-one-out sensitivity analysis revealed that none of the individual studies had a significant impact on the overall pooling result. In the subgroup analysis (Table 2), the pooled HR for BCR was 1.80 (95% CI 1.34–2.42) among patients receiving radical prostatectomy [11–14, 20–22], while 0.98 (95% CI 0.66–1.45) in those receiving radiotherapy [8–9].

**Table 2:** Subgroup analysis on biochemical recurrence

Subgroup	Number of study	Pooled HR	95% CI	Heterogeneity across studies
Treatment pattern				
RP Radiotherapy	7 2	1.80 0.98	1.34–2.42 0.66–1.45	*p* = 0.052; I^2^ = 51.9% *p* = 0.444; I^2^ = 0.0%
Sample sizes				
≥ 1000 < 1000	2 7	1.51 1.66	1.19–1.91 1.09–2.51	*p* = 0.653; I^2^ = 0.0% *p* = 0.005; I^2^ = 67.8%
Risk of patients				
High-risk Others	2 7	2.75 1.44	1.57–4.82 1.08–1.92	*p* = 0.696; I^2^ = 0.0% *p* = 0.030; I^2^ = 56.9%
Region				
Asia No-Asia	5 4	2.05 1.31	1.27–3.31 1.01–1.71	*p* = 0.029; I^2^ = 62.8% *p* = 0.235; I^2^ = 29.5%
Follow-up duration				
> 2 years ≤ 2 years	7 2	1.44 2.27	1.06–1.97 1.52–3.38	*p* = 0.028; I^2^ = 57.6% *p* = 0.787; I^2^ = 0.0%
Adjusted pretreatment PSA				
Yes No	7 2	1.41 2.39	1.06–1.89 1.59–3.59	*p* = 0.049; I^2^ = 52.5% *p* = 0.469; I^2^ = 0.0%

HR, hazard ratio; CI, confidence interval; RP, radical prostatectomy; PSA, prostate-specific antigen

### Publication bias

**Fig. 3:**
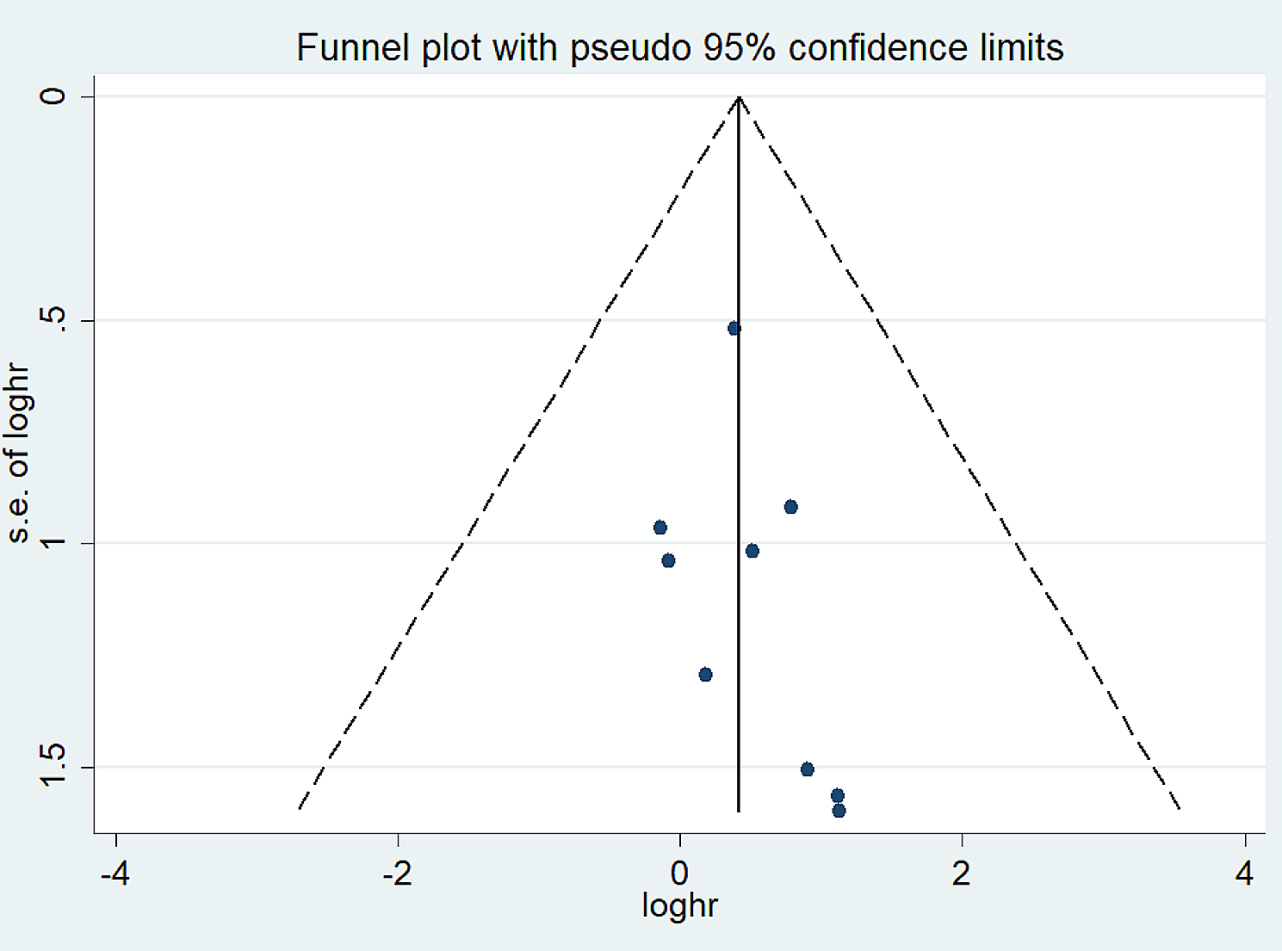
Publication bias determination using the funnel plots

There was no evidence of publication bias according to the Begg’s test (*p* = 0.175), Egger’s test (*p* = 0.368), and funnel plots (Fig. 3).

## Discussion

To the best of our knowledge, this is the first meta-analysis to assess the impact of pretreatment PSAD on BCR of prostate cancer after primary treatment. Our main finding suggests that elevated pretreatment PSAD may be an independent predictor for BCR of prostate cancer after radical prostatectomy. Compared to patients with low PSAD, those with high PSAD had an 80% higher risk of BCR after radical prostatectomy. However, there was no clear association between pretreatment PSAD and BCR of prostate cancer in the radiotherapy subgroup.

Apart from the categorical analysis of the PSAD, when considered as a continuous variable, was an independent risk factor for BCR in patients with intermediate-risk prostate cancer who underwent radical prostatectomy [23]. This association was also observed in patients with high-risk and very high-risk prostate cancer, even after adjusting for other factors [24]. These findings provide additional evidence for the predictive value of pretreatment PSAD in predicting BCR in prostate cancer patients.

One potential issue with the interpretation of the findings is the treatment pattern. Our subgroup analysis revealed that elevated PSAD was only a significant predictor of BCR in patients who underwent radical prostatectomy, but not in those who received radiotherapy. This suggests that PSAD may have a stronger predictive value after surgery compared to after radiotherapy. Additionally, the predictive value of PSAD seemed to be more pronounced in high-risk prostate cancer patients according to the D’Amico criteria. However, it should be noted that these findings were based on a small number of studies. Therefore, further research is needed to validate these results.

The prevalence of patients developing BCR was up to 55.1% in the included studies. Our meta-analysis provides some evidence to support elevated pretreatment PSAD was associated with an increased risk of BCR in prostate cancer patients. Incorporating PSAD into active surveillance protocols may improve the accuracy of predicting BCR in these patients. Additionally, elevated PSAD levels have been linked to high Gleason scores [6], advanced pathological stages [25], and extracapsular extension [12]. Therefore, assessing PSAD in prostate cancer patients has the potential to aid in clinical decision making.

Our meta-analysis has certain limitations that should be acknowledged. Firstly, all of the studies included in our analysis were retrospective designs, which carry inherent selection bias and may have unmeasured confounders. Secondly, the use of different cutoff values for PSAD in the included studies makes it difficult to apply our findings in a clinical setting. Thirdly, there was no uniform definition of BCR in the included studies. We only selected studies that reported at least two consecutive rises in PSA levels after curative treatment, and therefore, some studies that reported a single rise in PSA levels were not included in our analysis. However, these studies still provided valuable data on the association between PSAD and BCR in prostate cancer. Fourthly, there was significant heterogeneity in both the overall and subgroup analyses, which may be attributed to variations in patient and tumor characteristics, PSAD cutoff values, methods for measuring prostate volume, and treatment approaches. Fifthly, this meta-analysis was not prospectively registered in PROSPERO and other international databases. Finally, the number of patients included in our analysis was relatively small, particularly in some subgroups.

In conclusion, our analysis suggests that an elevated pretreatment PSAD may be an independent predictor for BCR of prostate cancer after radical prostatectomy. Therefore, incorporating the assessment of pretreatment PSAD may improve risk stratification for BCR in prostate cancer patients. However, it is important to note that our conclusion is limited by the fact that we only analyzed retrospective studies. Future high-quality prospective studies are required to validate the our findings.

### Electronic supplementary material

Below is the link to the electronic supplementary material.

Supplementary Material 1

Supplementary Material 2

## Data Availability

All data generated or analyzed during this study are included in this published article and its additional files.

## Abbreviations

PSAD: Prostate-specific antigen density
HR: Hazard ratios
CI: Confidence intervals
BCR: Biochemical recurrence
NOS: Newcastle-Ottawa Scale
