# Supplementary material for: Pretreatment prostate-specific antigen density as a predictor of biochemical recurrence in patients with prostate cancer: a meta-analysis
Source: BMC Cancer. 2024 Mar 6;24:305. doi: 10.1186/s12885-024-12029-8 (PMC10916317; doi:10.1186/s12885-024-12029-8)
Supplement: Supplementary file 1 — Supplementary Material 1 [file 12885_2024_12029_MOESM1_ESM.docx]

**Supplemental Text S1 – Search strategy**

| 1. **PubMed Search** | Query | Items found |
| --- | --- | --- |
| #1 | Search: **(((prostate neoplasms) OR (prostate cancer)) OR (prostate tumor)) OR (prostate carcinoma)** | 215,186 |
| #2 | Search: **(prostate-specific antigen density) OR (PSAD)** | 2,759 |
| #3 | Search: **((biochemical recurrence) OR (biochemical failure)) OR (relapse)** | 756,595 |
| #4 | Search: **((#1) AND (#2)) AND (#3)** | **253** |

| **2. Embase Search** | Query | **Items found** |
| --- | --- | --- |
| #1 | **Search** 'prostate specific antigen density'/exp OR 'prostate specific antigen density' OR (('prostate'/exp OR prostate) AND specific AND ('antigen'/exp OR antigen) AND ('density'/exp OR density)) | 4,597 |
| #2 | **Search** PSAD | 1,656 |
| #3 | **Search** #1 OR #2 | 5,252 |
| #4 | **Search** 'prostate neoplasms'/exp OR 'prostate neoplasms' OR (('prostate'/exp OR prostate) AND ('neoplasms'/exp OR neoplasms)) | 338,593 |
| #5 | **Search** 'prostate cancer'/exp OR 'prostate cancer' OR (('prostate'/exp OR prostate) AND ('cancer'/exp OR cancer)) | 350,831 |
| #6 | **Search** 'prostate tumor'/exp OR 'prostate tumor' OR (('prostate'/exp OR prostate) AND ('tumor'/exp OR tumor)) | 345,110 |
| #7 | **Search** 'prostate carcinoma'/exp OR 'prostate carcinoma' OR (('prostate'/exp OR prostate) AND ('carcinoma'/exp OR carcinoma)) | 89,871 |
| #8 | **Search** #4 OR #5 OR #6 OR #7 | 364,373 |
| #9 | **Search** 'biochemical recurrence'/exp OR 'biochemical recurrence' OR (biochemical AND ('recurrence'/exp OR recurrence)) | 24,926 |
| #10 | **Search** 'biochemical failure'/exp OR 'biochemical failure' OR (biochemical AND ('failure'/exp OR failure)) | 47,141 |
| #11 | **Search** 'relapse'/exp OR relapse | 329,583 |
| #12 | Search #9 OR #10 OR #11 | 385,784 |
| #13 | Search #3 AND #8 AND #12 | 338 |
| #14 | Search #13 AND ('article'/it OR 'article in press'/it) | **196** |

|  |  |  |
| --- | --- | --- |
| **3.** **Web of Science Search** | **Query** | **Items found** |
| #1 | Search (**prostate neoplasms** (Topic) or **prostate cancer** (Topic) or **prostate tumor** (Topic) or **prostate carcinoma** (Topic)) | 357,935 |
| #2 | Search (**prostate-specific antigen density** (Topic) or **PSAD** (Topic)) | 3,948 |
| #3 | **Search (biochemical recurrence** (Topic) or **biochemical failure** (Topic) or **relapse** (Topic)**)** | 392,945 |
| #4 | **Search #1 AND #2 AND #3 AND article (Publication type)** | **277** |
|  |  |  |
|  |  |  |
|  |  |  |
|  |  |  |
|  |  |  |
