# Supplementary material for: Pretreatment prostate-specific antigen density as a predictor of biochemical recurrence in patients with prostate cancer: a meta-analysis
Source: BMC Cancer. 2024 Mar 6;24:305. doi: 10.1186/s12885-024-12029-8 (PMC10916317; doi:10.1186/s12885-024-12029-8)
Supplement: Supplementary file 2 — Supplementary Material 2 [file 12885_2024_12029_MOESM2_ESM.doc]

~~Supplemental Table S1 Methodological quality of the included studies based on the Newcastle-Ottawa Scale.~~

| Author/year | Representativeness of the exposed cohort | Selection of the non-exposed cohort | Ascertainment of exposure | Demonstration that outcome was not present at study start | Comparability of cohorts based on the design or analysis | Assessment of outcome | Enough follow-up periods (≥2 years) | Adequacy of follow-up of cohorts | Total scores |
| --- | --- | --- | --- | --- | --- | --- | --- | --- | --- |
| Ingenito 1997 [8] | ★ | **★** | ★ | ★ | ★★ |  | ★ |  | 7 |
| Aref 1998 [9] | ★ | **★** | ★ | ★ | ★**★** |  | ★ | ★ | 8 |
| Busch 2012 [11] | ★ | **★** | ★ | ★ | ★★ |  | ★ | ★ | 8 |
| Gandaglia 2015 [12] |  | **★** | ★ | ★ | ★★ | ★ | ★ |  | 7 |
| Hashimoto 2015 [13] | ★ | **★** | ★ | ★ | ★ | ★ |  | ★ | 7 |
| Yashi 2017 [14] |  | ★ | ★ | ★ | ★ |  | ★ | ★ | 6 |
| Peng 2019 [20] |  | ★ | ★ | ★ | ★★ | ★ | ★ |  | 7 |
| Shida 2022 [21] |  | ★ | ★ | ★ | ★★ | ★ |  |  | 6 |
| Yan 2022 [22] | ★ | ★ | ★ | ★ | ★★ | ★ | ★ |  | 8 |
